# Hypoglycemia-activated Hypothalamic Microglia Impairs Glucose Counterregulatory Responses

**DOI:** 10.1038/s41598-019-42728-3

**Published:** 2019-04-17

**Authors:** Zsuzsanna Winkler, Dániel Kuti, Ágnes Polyák, Balázs Juhász, Krisztina Gulyás, Nikolett Lénárt, Ádám Dénes, Szilamér Ferenczi, Krisztina J. Kovács

**Affiliations:** 1 Laboratory of Molecular Neuroendocrinology, Institute of Experimental Medicine, Budapest, Hungary; 2 János Szentágothai Doctoral School of Neurosciences, Semmelweis University, Budapest, Hungary; 3 Laboratory of Neuroimmunology, Institute of Experimental Medicine, Budapest, Hungary

**Keywords:** Microglia, Hypothalamus

## Abstract

Glucose is a major fuel for the central nervous system and hypoglycemia is a significant homeostatic stressor, which elicits counterregulatory reactions. Hypothalamic metabolic- and stress-related neurons initiate these actions, however recruitment of glia in control such adaptive circuit remain unknown. Groups of fed- and fasted-, vehicle-injected, and fasted + insulin-injected male mice were compared in this study. Bolus insulin administration to fasted mice resulted in hypoglycemia, which increased hypothalamo-pituitary-adrenal (HPA) axis- and sympathetic activity, increased transcription of neuropeptide Y (*Npy*) and agouti-related peptide (*Agrp*) in the hypothalamic arcuate nucleus and activated IBA1+ microglia in the hypothalamus. Activated microglia were found in close apposition to hypoglycemia-responsive NPY neurons. Inhibition of microglia by minocycline increased counterregulatory sympathetic response to hypoglycemia. Fractalkine-CX3CR1 signaling plays a role in control of microglia during hypoglycemia, because density and solidity of IBA1-ir profiles was attenuated in fasted, insulin-treated, CX3CR1 KO mice, which was parallel with exaggerated neuropeptide responses and higher blood glucose levels following insulin administration. Hypoglycemia increased *Il-1b* expression in the arcuate nucleus, while IL-1a/b knockout mice display improved glycemic control to insulin administration. In conclusion, activated microglia in the arcuate nucleus interferes with central counterregulatory responses to hypoglycemia. These results underscore involvement of microglia in hypothalamic regulation of glucose homeostasis.

## Introduction

Blood glucose concentration is under strict physiological control. Glucose levels falling below 3,5 mM provoke centrally integrated bodily responses to mobilize glucose from peripheral stores and increase gluconeogenesis aiming at restoration of normoglycemia^1^. Due to impaired autonomic counterregulatory responses (CRR), a serious condition, hypoglycemia associated autonomic failure (HAAF), may occur in diabetic patients^2^.

Glucose concentration is monitored by multiple peripheral and central glucosensors in the hypothalamus and brainstem^3^. These neurons are found in close surrounding of circumventricular organs, i.e. the median eminence and the area postrema, through which additional metabolic-related hormonal- and nutritional signals are received^4^. Information in glucoregulatory control circuits then relayed to autonomic pre-ganglionic and neuroendocrine motor neurons, which regulate effectors such as chromaffin cells in the adrenal medulla, α and β cells in pancreatic islets and corticotropes/somatotropes in the anterior pituitary^1^. Hypoglycemia, as a potential life threatening condition, results activation of stress-related neurocircuit, which shares some common effectors involved in glucose counterregulatory responses (sympatho-adrenal activation and glucocorticoid secretion)^5^.

Metabolic- and stress-related circuits are intimately connected with immune regulation. Microglial cells are local representatives of the innate immune system in the CNS, which might be recruited in physiological responses to homeostatic challenges^6,7^. Microglia continuously screen tissue microenvironment via highly motile fine branches and become rapidly activated in response to subtle changes in synaptic activity or danger (damage)- and pathogen-associated molecular patterns (DAMPs and PAMPs)^8,9^.

Increasing evidence support the involvement of glial elements in regulation, shaping and modulation adaptive physiological responses^7^. Along these lines, the aim of the present study was to investigate changes of hypothalamic microglia in response to a strong homeostatic/metabolic challenge, hypoglycemia and to reveal the role of microglia in control of counterregulatory responses.

## Results

### Insulin injection to fasted mice results in hypoglycemia, activates hypothalamo-pituitary-adrenocortical (HPA) axis and increases adrenaline and glucagon secretion

**Figure 1:**
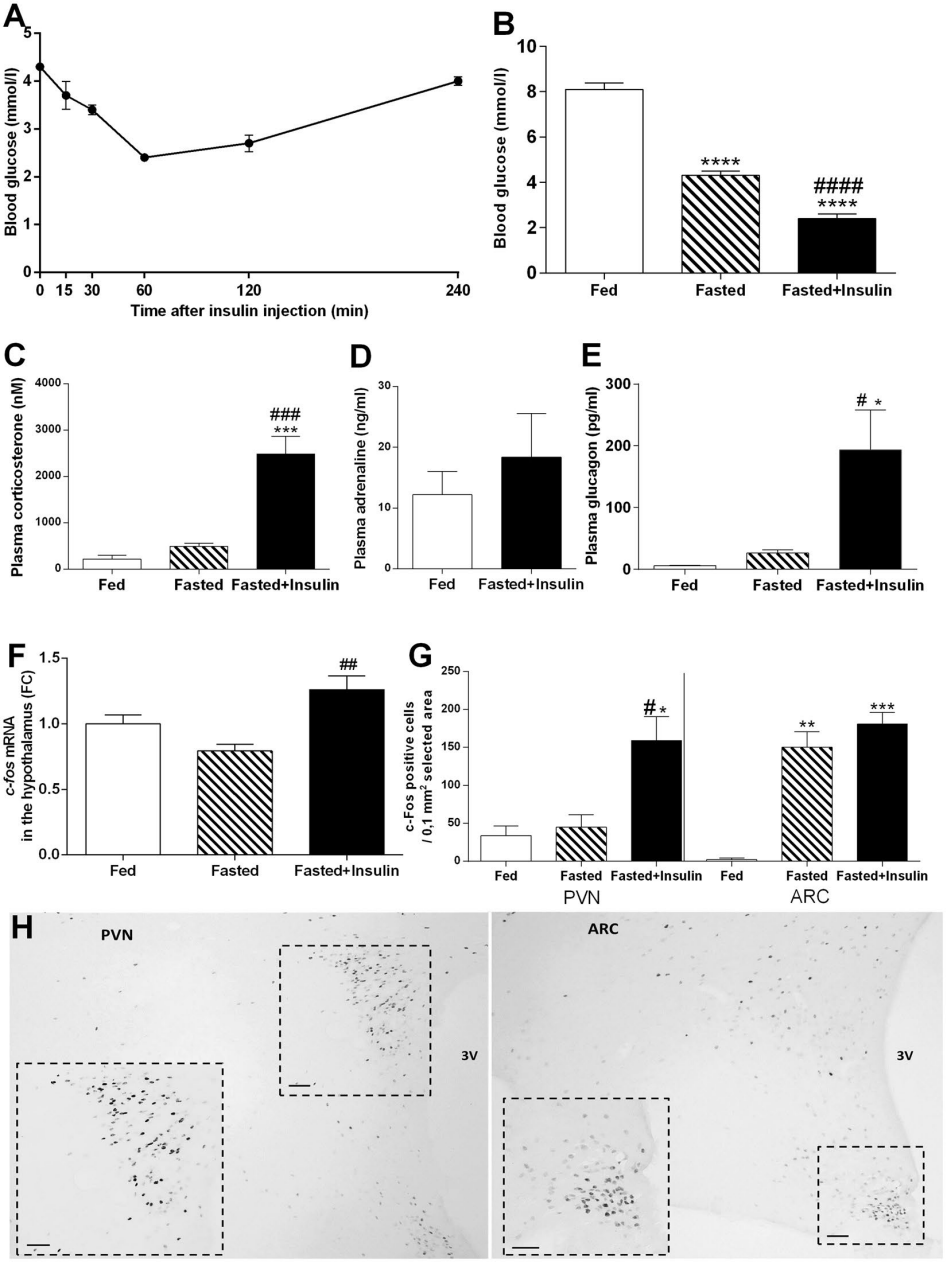
Blood glucose levels, counterregulatory hormones, and neuronal activation in hypothalamus after overnight fasting and insulin-induced hypoglycemia in C57BL/6 mice. (**A**) Time course of blood glucose levels in fasted mice following i.p. insulin injection (1IU/kg) (n = 12 per blood sampling time). (**B**) Blood glucose levels in fed, fasted and fasted + insulin treated groups 1 hour after i.p. saline/insulin injection (n = 29–33 per group). (**C**–**E**) Hormonal responses to fasting and insulin-induced hypoglycemia. Elevation of plasma corticosterone (**C**) (n = 3–4), adrenaline (**D**) (n = 4-4) and glucagon (**E**) (n = 5–8 per group) plasma concentrations 1 h after insulin challenge. (**F**) Relative expression level of *c-fos* mRNA as measured by qRT-PCR in hypothalamus of fed, fasted, and fasted + insulin treated mice (n = 4 per group). (**G**) Counts of c-FOS-ir neurons in hypothalamic paraventricular (PVN) and arcuate (ARC) nuclei in fasted and fasted + insulin injected mice (n = 3 per group). (**H**) Representative photographs illustrating c-FOS expression in PVN and ARC after insulin-induced hypoglycemia. Scale bars, 50 µm; 3 V, third ventricle. Fed: fed, saline-injected group; Fasted: O/N fasted, saline-injected group; Fasted + insulin: O/N fasted, insulin-injected group. Data are expressed as mean ± SEM. *p < 0.05, **p < 0.01, ***p < 0.001, ****p < 0.0001 vs. fed; ^#^p < 0.05, ^##^p < 0.01, ^###^p < 0.001, ^####^p < 0.0001 vs. fasted.

Single i.p. insulin injection to fasted C57BL/6 mice gradually decreased blood glucose levels to the 2.4 ± 0.2 mM minimum (hypoglycemia) at 60 min post-injection, then returned to the fasted baseline level by 240 min 4.02 ± 0.19 mM (Fig. 1A).

Based on this time course, all other measurements and comparisons were made at the 60 min time point.

One way ANOVA [F (2, 89) = 152.1, p < 0.0001] revealed significant difference between fed + vehicle; fasted + vehicle-injected and fasted + insulin treated groups at 60 min post-injection (Fig. 1B). Blood glucose levels in fed, vehicle-injected mice were 8.1 ± 0.29 mM, which decreased to 4.3 ± 0.2 mM after overnight fasting (Fig. 1B).

As a strong homeostatic stressor, hypoglycemia resulted in activation of HPA axis compared to fed [F (2, 6) = 67.93, p < 0.0001; Bonferroni’s post hoc test, t(6) = 10.71, p < 0.001] and fasted [F (2, 6) = 67.93, p < 0.0001; Bonferroni’s post hoc test, t(6) = 8.3, p < 0.001] state as indicated by elevated corticosterone concentration (Fig. 1C). Hypoglycemia increased plasma adrenaline levels, although the difference between insulin treated (18.35 ± 3.58 ng/ml) vs. vehicle-injected groups (12.19 ± 1.92 ng/ml) was not statistically different (p > 0.05) (Fig. 1D). Hypoglycemia resulted in a marked elevation of plasma glucagon levels compared to vehicle-injected, fed control [F (2, 18) = 6.22, p = 0.009; Bonferroni’s post hoc test, t(18) = 4.66, p < 0.05] and fasted mice [Bonferroni’s post hoc test, t(18) = 3.64, p < 0.05](Fig. 1E).

### Hypoglycemia activates stress-related and orexigenic neurons in the hypothalamus

Overnight fasting did not elevate *c-fos* mRNA levels in the whole hypothalamus compared to that of fed controls, while acute insulin-induced hypoglycemia significantly induced *c-fos* mRNA levels [F (2, 8) = 9.14, p = 0.009; Bonferroni’s post hoc test, t(8) = 4.27, p < 0.001] as measured by qPCR (Fig. 1F). Compared to fed and fasted controls, c-FOS induction was detected in the hypothalamic arcuate-, paraventricular nuclei (Fig. 1H) and in the lateral hypothalamic area (Supplementary Fig. 4) in fasted, insulin-injected mice. Quantitative analysis of c-FOS positive cell nuclei confirmed significant hypoglycemia-induced increase in the paraventricular nucleus compared to fed [F (2, 6) = 10.05, p = 0.01; Bonferroni’s post hoc test, t(6) = 5.73, p < 0.05] and fasted mice [Bonferroni’s post hoc test, t(6) = 5.22, p < 0.05] (Fig. 1G,H). In the arcuate nucleus, fasting [F (2, 6) = 41.56, p < 0.001; Bonferroni’s post hoc test, t(6) = 7.06, p < 0.01] and fasting plus insulin treatment increased the number of c-FOS positive profiles compared to fed controls [Bonferroni’s post hoc test, t(6) = 8.53, p < 0.001] (Fig. 1G,H).

**Figure 2:**
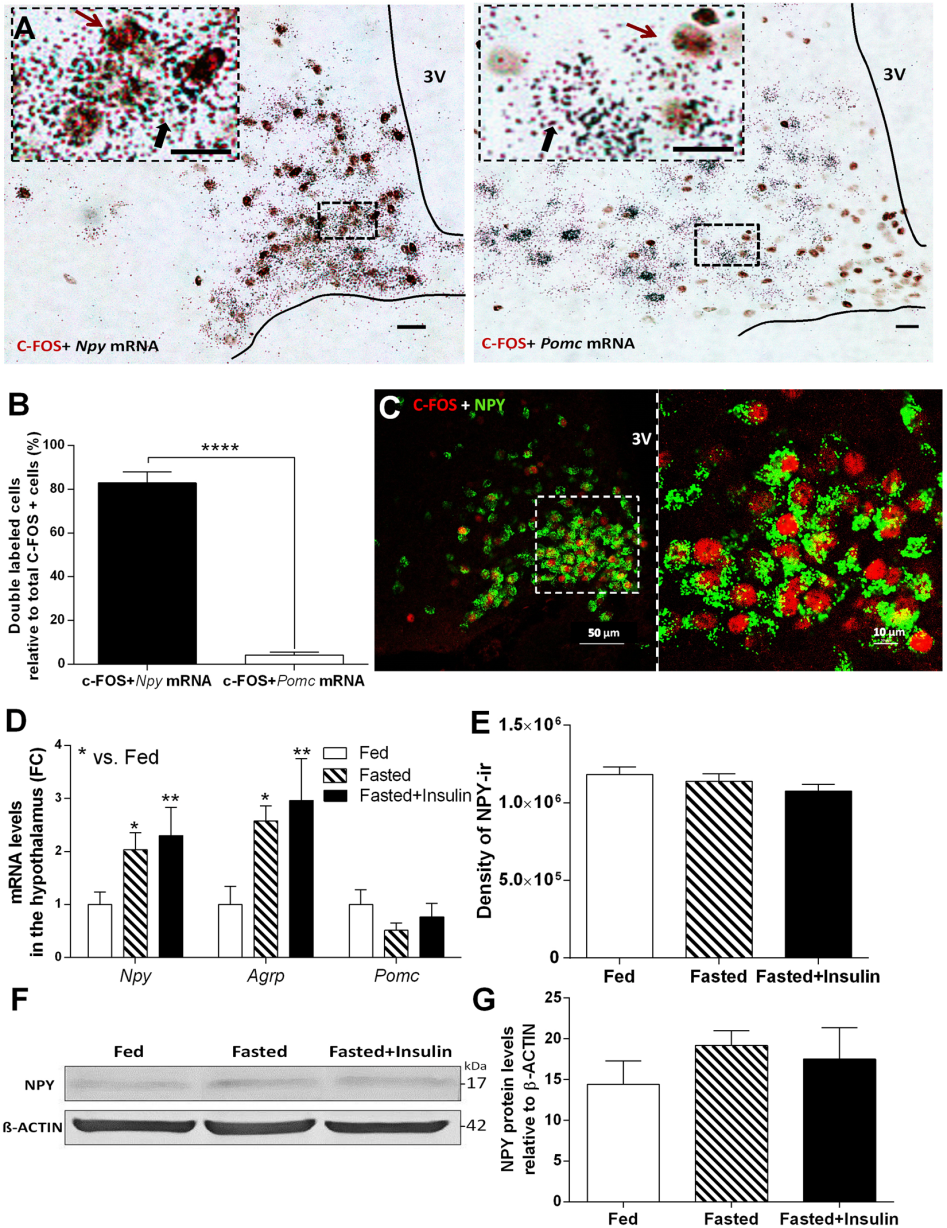
Hypoglycemia selectively activates orexigenic, NPY/AGRP -expressing neurons in hypothalamic arcuate nucleus (ARC). (**A**) Brightfield images of combined immunohistochemical (c-FOS; brown cell nuclei) and *in situ* hybridization histochemical [*Npy* or *Pomc* mRNA (black autoradiographic grains)] ARC preparations of fasted mice 1 h after insulin injection. Scale bars, 10 µm. (**B**) Colocalization of *Npy* or *Pomc* mRNA with c-FOS protein in ARC after insulin-induced hypoglycemia (n = 6–7). (**C**) Representative images of c-FOS (red) and NPY protein (green) colocalization in ARC of NPY-Ires-Cre ZsGreen reporter mice following insulin-induced hypoglycemia. (**D**) Expression of *Npy*, *Agrp* and *Pomc* mRNA in arcuate nucleus of fasted mice 1 h after insulin injection compared to fed and fasted, saline injected controls (n = 4 per groups). Fold change (FC) of mRNA expression was assessed by quantitative RT-PCR (qRT-PCR). (**E**) Quantitative analysis of NPY-immunostained hypothalamic sections of C57BL/6 mice. Bar graph represents the density of NPY-immunreactivity (ir) in the unit area of arcuate nucleus (n = 3 per groups). (**F**,**G**) NPY levels, representative Western blot image (**F**) and quantification (**G**) from hypothalamus of fed, fasted and fasted + insulin injected mice (n = 3-3 per group). Full-length blot is presented in Supplementary Fig. 3. Fed: fed, saline-injected group; Fasted: O/N fasted, saline-injected group; Fasted + insulin: O/N fasted, insulin-injected group. All data expressed as mean ± SEM. *p < 0.05, **p < 0.01 vs. fed. 3 V: third ventricle.

In order to reveal neuropeptide phenotype of hypoglycemia-activated neurons in the arcuate nucleus, double immune- and hybridization histochemical labeling approach was used (Fig. 2A). Combined *in situ* localization of *Npy* or *Pomc* mRNA with the activation marker, c-FOS in the medial basal hypothalamus revealed selective activation of orexigenic NPY neurons [t(11) = 38.86, p < 0.001]. 83% of all activated (c-FOS) positive neurons contained *Npy* mRNA, while only 4% of activated neurons expressed *Pomc* mRNA (Fig. 2B) in response to hypoglycemia. c-FOS and NPY protein colocalization was confirmed on brain sections from NPY-Ires-Cre ZsGreen reporter mice following overnight fasting and 1 h insulin injection (Fig. 2C).

Overnight fasting and fasting + insulin administration resulted in elevation of *Npy* [F (2, 8) = 9.93, p < 0.01] and *Agrp* mRNA levels in the hypothalamus [F (2, 8) = 12.01, p < 0.01]. As measured by RT-qPCR, *Pomc* mRNA expression remained unchanged between the groups (Fig. 2D).

Next, we checked if transcriptional changes in arcuate *Npy* neurons are translated to NPY protein. We did not find differences in density of NPY-immunoreactivity in the arcuate region of fed, fasted and fasted plus insulin-treated animals at 1 hour time point (Fig. 2E, Supplementary Fig. 2). Western blot analysis revealed a trend for increased hypothalamic NPY content in fasted and fasted + insulin treated mice (Fig. 2F,G, Supplementary Fig. 3).

Overnight fasting and insulin induced-hypoglycemia resulted in coexpression c-FOS and OREXIN-A in perifornical part of the lateral hypothalamus. Changes in *Orexin-a* mRNA levels (Relative fold change values: 0,99 ± 0,17 in fasted-; 0,70 ± 0,07 in fasted + insulin) and number of OREXIN-A-ir cells (Supplementary Fig. 4) were not significant. MCH neurons in the lateral hypothalamus did not express c-FOS at any of these conditions (Supplementary Fig. 4).

### Hypoglycemia results in morphological changes in hypothalamic microglia

**Figure 3:**
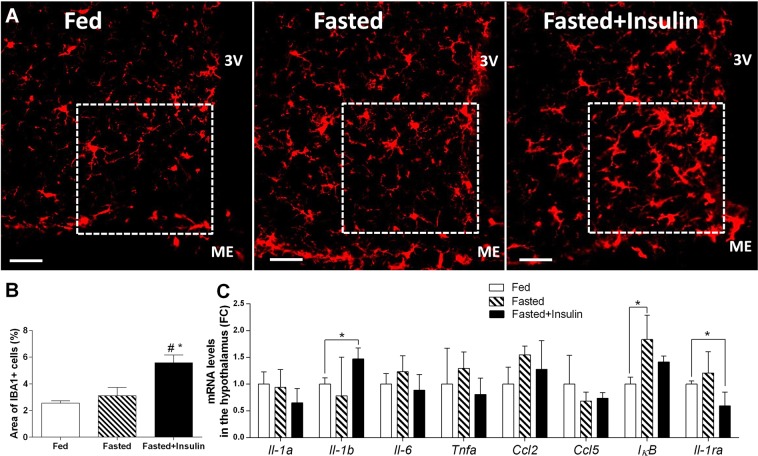
Hypoglycemia recruits microglia in the hypothalamus. (**A**) Representative photomicrographs, showing ionized calcium-binding adaptor molecule 1 (IBA1) immunoreactive (-ir) microglia in ARC of fed, fasted and fasted + insulin-injected mice. Scale bars, 20 µm. 3 V: third ventricle, ME: median eminence. (**B**) Mean ± SEM percentages of the area covered by IBA1-immunoreactivity per unit area in ARC (n = 3 per group). ROIs are shown on images. (**C**) Mean ± SEM values of relative mRNA levels of proinflammatory cytokines (*Il-1a Il-1b*, *Il-6*, *Tnfa*) chemokines (*Ccl2*, *Ccl5*) and *IκB*, *Il-1ra* in hypothalamus (n = 4 per groups). Fed: fed, saline-injected group; Fasted: O/N fasted, saline-injected group; Fasted + insulin: O/N fasted, insulin-injected group. All data on the figure expressed as mean ± SEM. *p < 0.05 vs. fed; ^#^p < 0.05 vs. fasted as determined by Bonferroni post hoc test.

Next, we investigated if hypoglycemia activates microglia in the hypothalamus, using ionized calcium-binding adaptor molecule 1 (IBA1) as microglia marker. In fed- and fasted, vehicle injected control animals, IBA1 immunostaining revealed predominantly resting form of microglia with moderate perisomatic immunoreactivity and fine branching processes (Fig. 3A). By contrast, in the medial basal hypothalamus of fasted plus insulin-injected, hypoglycemic animals, microglia displayed activated phenotype with thickened branches and increased IBA1-ir (Fig. 3A). Analysis of IBA1 positive cells in arcuate nucleus revealed an increase (Fig. 3B) in hypoglycemic C57BL/6 mice compared to fed [F (2, 6) = 10.17, p < 0.05; post hoc test, t(6) = 4.24, p < 0.05], and fasted, vehicle treated mice [F (2, 6) = 10.17, p < 0.05; post hoc test, t(6) = 3.45, p < 0.05]. Hypoglycemia-induced microglia activation was confined to the arcuate nucleus: qualitative and quantitative analysis of IBA1 positive profiles in the hypothalamic paraventricular nucleus (Supplementary Fig. 5), hippocampus and cortex (Supplementary Fig. 6) did not reveal morphological signs of activation.

We next measured hypothalamic expression of select proinflammatory markers, which are related to activated microglia. Among these, hypothalamic expression of *Il-1b* mRNA was increased in hypoglycemic mice, compared to fed animals [t(5) = 3.54, p < 0.05]. mRNA levels of *Il-1a*, *Il-6*, *Tnfa*, *Ccl2* (*Mcp-1*) and *Ccl5* (*Rantes*) remained unchanged 1 hour after insulin (Fig. 3C). *Iκb* mRNA level was significantly elevated in the hypothalamus of fasted animals compared to fed [F (2, 8) = 7.02, p = 0.02; Bonferroni’s post hoc test, t(8) = 3.73, p < 0.05], but not to hypoglycemic (fasted + insulin injected) mice (Fig. 3C). Furthermore, *Il-1ra* mRNA level was decreased in fasted + insulin injected mice compared to fed animals [F (2, 8) = 4.58, p = 0.05; t(5) = 2.61, p < 0.05] (Fig. 3C).

### Activated microglia make close appositions with c-FOS positive NPY neurons in the arcuate nucleus

**Figure 4:**
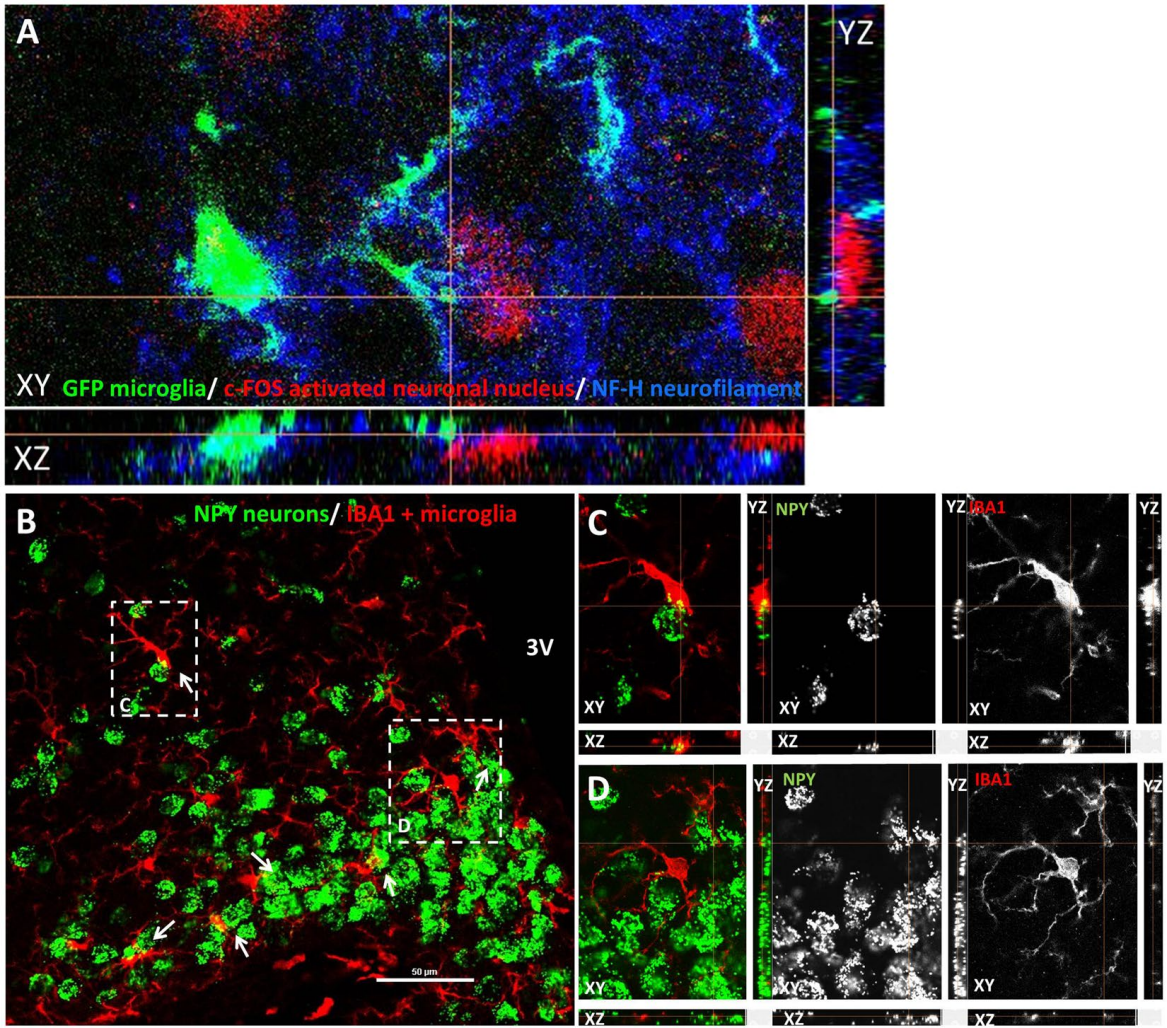
Microglia make close appositions with hypoglycemia-activated neurons in arcuate nucleus. (**A**) Confocal imaging of triple fluorescent-labeled section from ARC of hypoglycemic CX_3_CR1 ^+/gfp^ microglia reporter mouse. GFP (green) –microglia; c-FOS (red) –cell nuclei of activated neurons; and neurofilament H (NF-H) (blue) – neurons. (**B**) Representative images of contacts between NPY neurons (green) and IBA1 positive microglia (red) in hypoglycemic NPY-Ires-Cre ZsGreen reporter mouse line. The appositions between microglia and NPY neurons were revealed in the arcuate nucleus by using merged confocal Z-stack images with orthogonal views XZ and YZ, at 60x magnification. White arrows mark close appositions between microglia and NPY neurons.

Next, we investigated relation of microglia to hypoglycemia-responsive neurons in the arcuate nucleus. The “thickened” microglial cells were recruited around c-FOS+, activated neuron population in the rostral and medial portion of the arcuate nucleus. Confocal imaging of triple fluorescent labeled material in fasted, insulin-injected CX_3_CR1^+/gfp^ mice (microglia reporter strain on C57BL/6 background) revealed close apposition of microglia and hypoglycemia-activated, c-FOS positive neurons (Fig. 4A). Furthermore, similar contacts were found between NPY neurons and IBA1 positive microglia in hypoglycemic NPY reporter (NPY-Ires-Cre ZsGreen) mouse line (Fig. 4B).

### Minocycline attenuates microglia activation in response to insulin and results in less severe hypoglycemic response

**Figure 5:**
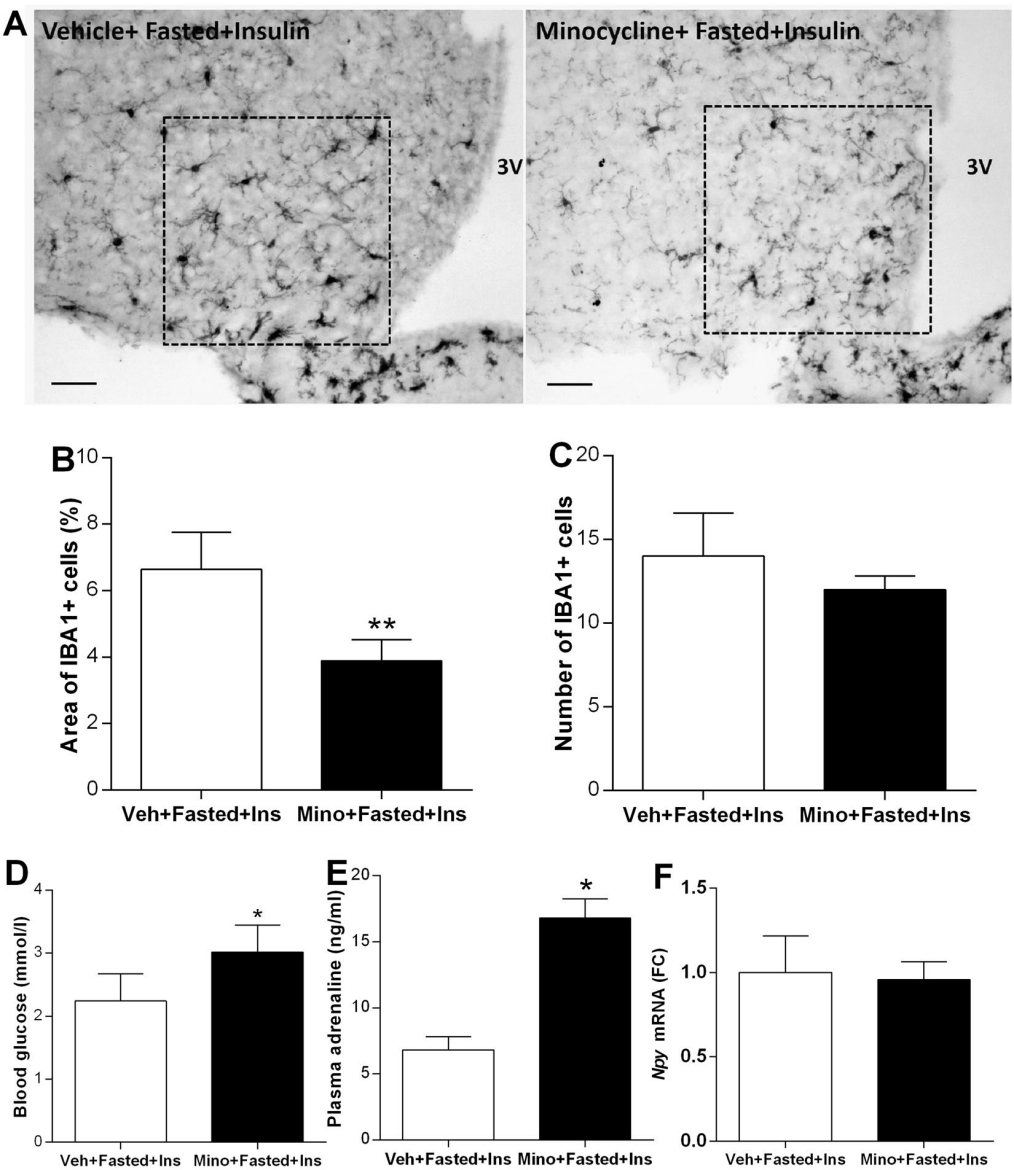
Manipulation of microglia affects glycemia and counterregulatory response to hypoglycemia. (**A**) Photomicrographs showing IBA1-ir microglia in arcuate nucleus of vehicle- or minocycline treated, fasted mice 1 h following insulin administration. Scale bar: 20 µm; 3V-third ventricle. (**B**) Area covered by IBA1-ir profiles expressed as percentage of the unit arcuate area (n = 5-5). (**C**) Number of IBA1-ir ARC cells in fasted + insulin injected mice, pretreated i.c.v. vehicle or minocycline (n = 5-5). (**D**) Blood glucose levels of fasted + insulin-treated mice, which were pretreated either with minocycline or vehicle i.c.v. Note that inhibition of microglia activity attenuated the drop of blood glucose following insulin injection (n = 5-5). (**E**) Plasma adrenaline levels in minocycline or vehicle-pretreated, fasted animals following insulin treatment (n = 3-3). (**F**) Relative hypothalamic *Npy* mRNA expression in minocycline or vehicle-pretreated animals following O/N fasting and insulin administration, measured by qRT-PCR (n = 3-3). All data expressed as mean ± SEM. In all graphs, *p < 0.05, **p < 0.01 (two-sided t-test).

Next, we investigated whether inhibition of microglia affects glycemic response to insulin in fasted animals. Intracerebroventricular (i.c.v.) infusion of minocycline, an anti-inflammatory drug that impacts on microglial reactivity, attenuated insulin-induced microglia activation in the hypothalamus of fasted mice (Fig. 5A). The area of IBA1 positive cells was significantly smaller in the arcuate nucleus of minocycline treated, fasted and insulin-injected mice compared to fasted, vehicle-injected controls [t(6) = 4.33, p < 0.01] (Fig. 5B). Number of IBA1 positive profiles in the arcuate nucleus was reduced following minocycline administration, however, the difference between vehicle-treated vs minocycline-treated animals was not significant (Fig. 5C). In fasted mice, microglia inhibition by minocycline resulted in 36% higher blood glucose level 1 h after insulin [t(8) = 2.87, p < 0.05] (Fig. 5D) compared to vehicle-injected, insulin-treated controls. Insulin-induced plasma adrenaline level was higher in minocycline-treated animals than in vehicle-treated mice [t(6) = 5.62, p < 0.05] (Fig. 5E). *Npy* mRNA expression in ARC, as measured by qRT-PCR, was not different (Fig. 5F).

Minocycline administration did not significantly affect resting appearance of microglia in PVH, LH, hippocampus and cortex (Supplementary Fig. 6).

### Fractalkine receptor (CX_3_CR1) deficient mice mount increased counterregulatory responses to insulin-induced hypoglycemia and neuroglycopenia

**Figure 6:**
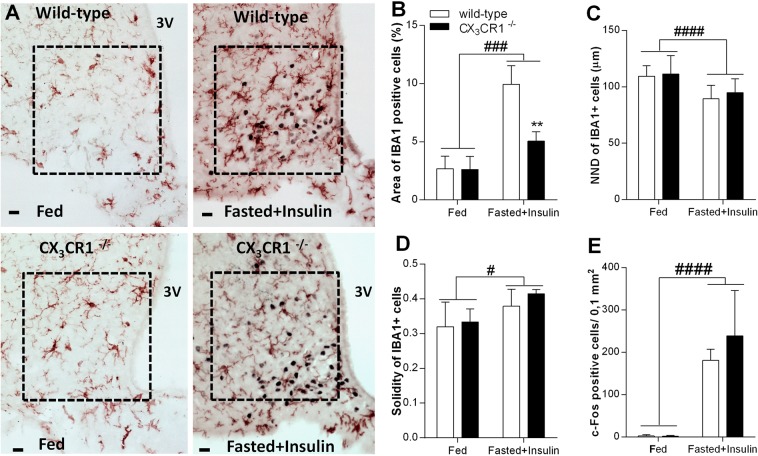
Neuron-microglia communication is critical for microglia activation in insulin-induced hypoglycemia. (**A**) Photomicrographs showing c-FOS (black cell nuclei) and IBA1-ir profiles (brown) in arcuate nucleus of wild-type and fractalkine receptor deficient (CX_3_CR1^−/−^), fed + vehicle treated and fasted + insulin-treated mice. Note microglia accumulation close to c-FOS expressing neurons in wild-type, fasted + insulin treated animals. Scale bar: 20 µm; 3 V: third ventricle. (**B–D**) Quantitative analysis of microglia population in arcuate nucleus (n = 3 per groups). (**B**) Area % occupied by IBA1 positive profiles; (**C**) Nearest neighbor distance; (**D**) Solidity index; (**E**) Number of c-FOS positive cells in arcuate nucleus of wild-type and CX_3_CR1^−/−^, fed or fasted + insulin-treated mice (n = 3 per group). Mean ± SEM values. Two way ANOVA: significant treatment effect indicated by ^#^p < 0.05, ^###^p < 0.001, ^####^p < 0.0001 and genotype effect indicated by **p < 0.01. Fed: fed, saline-injected group; Fasted + Insulin: O/N fasted, insulin-injected group.

Fractalkine receptor is critically implicated in activation of microglia in response to physiological and traumatic insults. Next, we compared effects of insulin injections to fasted, wild type and fractalkine receptor deficient mice. In these animals, second exon of the *Cx3cr1* gene has been replaced by *Egfp* (CX_3_CR1 ^gfp/gfp^ = CX_3_CR1^−/−^ on C57BL/6 background^10^). Qualitative and quantitative analysis of IBA1 positive microglia revealed significant effects of insulin treatment in the ARC (Fig. 6A–E). Area covered by IBA1 immuno-labelled profiles (Fig. 6B) [F (1, 8) = 49.88, p < 0.001], and solidity of IBA1 positive cells increased significantly (Fig. 6D) [F (1, 8) = 6.62, p = 0.03]. Insulin treatment had no effect on the number of microglial cells. Bonferroni’s multiple comparison test indicated significantly smaller area occupied by IBA1 immuno-reactive cells (Fig. 6B) in ARC of fasted, insulin treated, fractalkine receptor deficient animals, than in similarly treated, C57BL/6 animals [t(8) = 5.03, p < 0.05]. The nearest neighbor distance between microglial cells in the ARC decreased significantly after insulin injection [F (1, 43) = 20.6, p < 0.0001] (Fig. 6C). C-FOS immunostaining revealed a trend for increased neuronal activation in hypoglycemic CX_3_CR1^−/−^ mice compared to wild-type mice (Fig. 6E).

**Figure 7:**
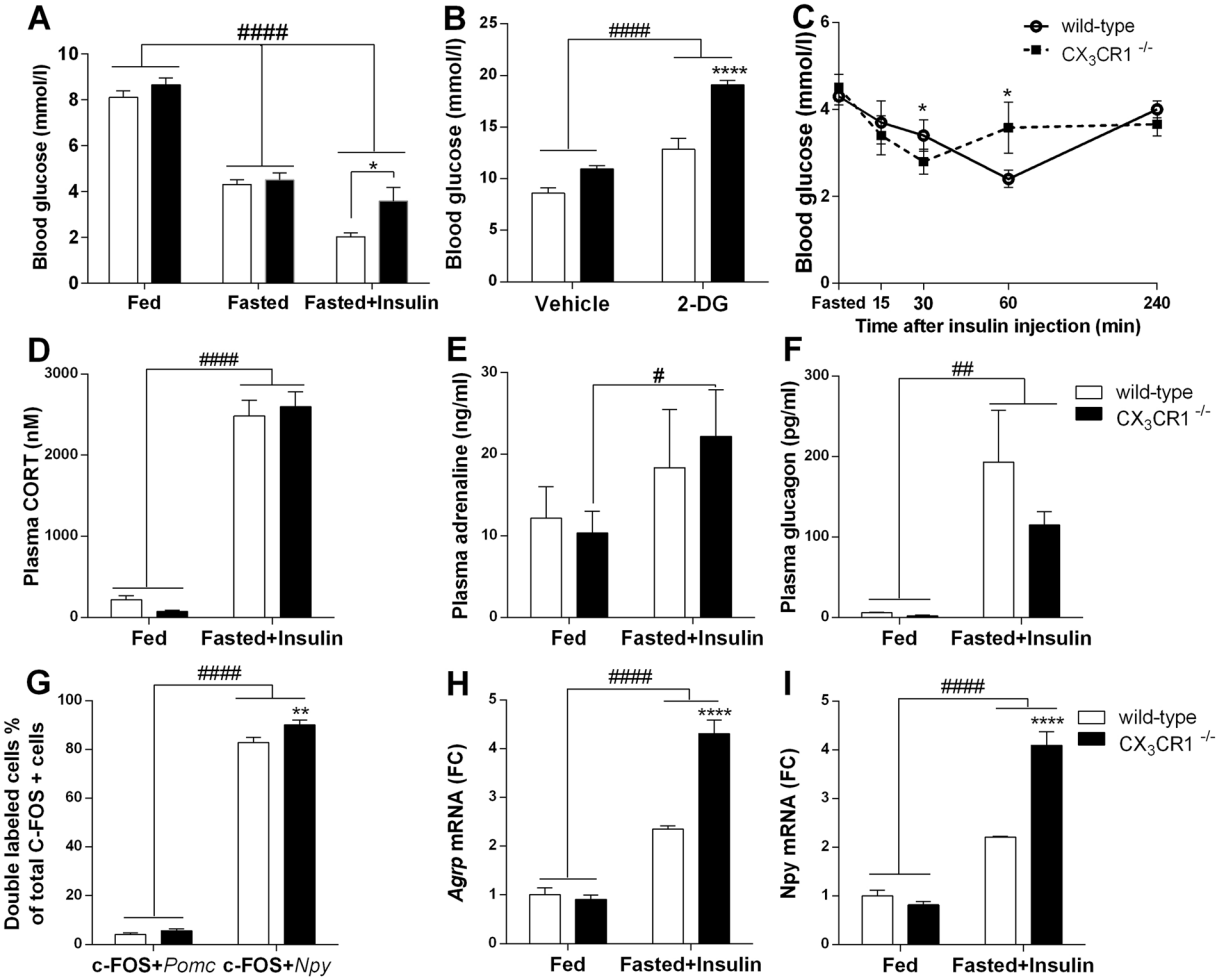
Neuron-microglia communication through fractalkine receptor is critical in organization of counterregulatory response to insulin-induced hypoglycemia and 2-deoxy glucose-induced neuroglycopenia (**A**) Blood glucose levels in fed, fasted, fasted + insulin-injected, wild-type and fractalkine receptor deficient (CX_3_CR1^−/−^) mice (n = 5–20). (**B**) Comparison of blood glucose responses in fed, wild-type and CX_3_CR1^−/−^ mice to neuroglycopenia induced by i.p. injection of 2-deoxyglucose (2-DG, 200 mg/kg) or vehicle. (**C**) Blood glucose levels in response to i.p. insulin injection (1IU/ml/kg bw) during insulin tolerance test (n = 12 per groups) (**D–F**) Mean ± SEM values of plasma corticosterone (**D**); adrenaline (**E**) and glucagon (**F**) levels in fed and fasted + insulin-treated, wild-type and CX_3_CR1^−/−^ mice (n = 3–8). (**G**) Quantitative analysis of hypoglycemia-responsive *Npy* and *Pomc* neurons. Values obtained from fasted + insulin-injected, wild- type and CX_3_CR1^−/−^ mice (n = 6–8) and expressed as percentage of double labeled cells relative to all c-FOS positive neurons in the arcuate nucleus. (**H**,**I**) Hypothalamic *Agrp* and *Npy* mRNA levels in fed and fasted + insulin-treated, wild-type and CX_3_CR1^−/−^ mice (n = 4–4) as measured by qRT-PCR. Two-way ANOVA followed by Bonferroni’s post-hoc test revealed significant treatment effect indicated by ^#^p < 0,05, ^##^p < 0,01, ^####^p < 0,0001 or genotype effect indicated by *p < 0.05, ****p < 0.0001. Fed: fed, saline-injected group; Fasted: O/N fasted, saline-injected group; Fasted + insulin: O/N fasted, insulin-injected group.

Wild-type and CX_3_CR1^−/−^ mice displayed similar blood glucose levels in fed and fasted states, however fasted CX_3_CR1^−/−^ mice did not develop hypoglycemia in response to insulin [blood glucose in C57BL/6: 2,02 ± 0,13 mM vs. CX_3_CR1^−/−^: 3,58 ± 0,45 mM; t(8) = 2.54, p = 0.035] (Fig. 7A).

Systemic administration of 2-deoxy-D-glucose (2-DG) resulted in counterregulatory increase in blood glucose levels in fed mice [F (1, 11) = 80.19, p < 0.0001]. 1 h following 2-DG, plasma glucose levels in CX_3_CR1^−/−^ animals were significantly higher than those of C57BL/6 mice [F (1, 11) = 38.83, p < 0.0001; Bonferroni’s post hoc test, t(11) = 6.65, p < 0.0001] (Fig. 7B).

To rule out the possibility of insulin insensitivity in CX_3_CR1^−/−^ mice, we performed insulin tolerance tests in both genotypes. Initial decline of glucose levels in fasted animals following insulin injection (Fig. 7C) and the area under the glucose curve (AUC) did not reveal significant differences in insulin sensitivity between wild type and CX_3_CR1^−/−^ mice (AUC: C57BL/6: 982 mM/240 min vs. CX_3_CR1^−/−^ :1044 mM/240 min). We did not detect significant differences in glucose tolerance between the two genotypes (Supplementary Fig. 7).

Fractalkine receptor deficient mice mounted significantly increased plasma adrenaline response following insulin compared to vehicle-treated fed controls [F (1, 10) = 9.98, p = 0.01; Bonferroni’s post hoc test, t(10) = 2.75, p < 0.05] (Fig. 7E). However, genotype did not influence hypoglycemia-induced plasma corticosterone [F (1, 11) = 0.01, p = 0.93] (Fig. 7D), and glucagon responses [F (1, 23) = 1.05, p = 0.32] (Fig. 7F).

As shown on Fig. 7H,I fasted, insulin-treated, CX_3_CR1^−/−^ mice displayed significantly higher hypothalamic *Npy* [F (1, 12) = 30.28, p < 0.001; Bonferroni’s post hoc test, t(12) = 8.60, p < 0.001] and *Agrp* [F (1, 12) = 31.61, p < 0.001; Bonferroni’s post hoc test, t(12) = 8.34, p < 0.001] mRNA response than wild-type animals. *Pomc* levels in ARC remained unchanged in both genotypes. Co-localization of c-FOS immunoreactivity with *Npy* or *Pomc* mRNA revealed preferential activation of NPY neurons. Furthermore, higher number of double stained profiles was found in fasted insulin treated, CX_3_CR1^−/−^ animals compared to fasted, insulin treated wild-type [F (1, 23) = 7.87, p = 0.01; Bonferroni’s post hoc test, t(23) = 3.33, p < 0.001] (Fig. 7G).

**Figure 8:**
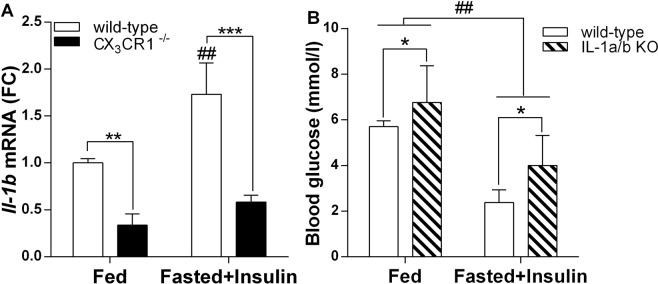
Proinflammatory cytokine IL-1 is associated with insulin-induced hypoglycemia. (**A**) Relative quantities of hypothalamic *Il-1b* mRNA of fed and fasted + insulin-injected, wild-type and CX_3_CR1^−/−^ mice (n = 3 per groups). (**B**) IL-1 a/b knockout animals do not display severe hypoglycemia in response to insulin challenge as do wild type animals. Mean ± SEM blood glucose levels in fed and fasted + insulin treated, wild-type and IL-1 a/b KO mice, 1 h after i.p. injections (n = 3–5). Two-way ANOVA with Bonferroni post hoc test revealed main treatment effect of ^##^p < 0.01; main genotype effect: *p < 0.05, **p < 0.01, ***p < 0.001. Fed: fed, saline-injected group; Fasted + Insulin: O/N fasted, insulin-injected group.

Danger signals provoke release of proinflammatory cytokines, such as IL-1 from activated glial cells. We found increased *Il-1b* mRNA level in hypothalamic blocks of control, C57BL/6 mice in response to insulin-induced hypoglycemia [F (1, 8) = 21.60, p < 0.01; Bonferroni’s post hoc test, t(8) = 4.93, p < 0.001]. By contrast, hypoglycemia-induced increase of *Il-1b* mRNA was not seen in mice with impaired fractalkine signaling between neurons and microglia [Bonferroni’s post hoc test, t(8) = 1.65, p > 0.05]. (Fig. 8A).

To assess the impact of IL-1 in organization of glucose responses hypoglycemia, IL-1 a/b KO mice were challenged with insulin. We found that IL-1 a/b KO mice displayed higher blood glucose concentration in response to insulin administration than wild type controls [main effect of genotype: F(1,12) = 5.02, p = 0.045], their blood glucose concentration did not decrease below 3 mM (Fig. 8B). In response to 2-DG, IL-1 a/b KO mice have higher glucose levels (18.04 ± 2.23 mM) compared to wild type controls (14.70 ± 2.03 mM), however the difference between 2-DG-treated groups was not significant (data not shown).

## Discussion

Here we show that insulin-induced hypoglycemia selectively activates microglia in the hypothalamic arcuate nucleus and this microglial activation likely attenuates full expression of counterregulatory responses to hypoglycemia.

Hypoglycemia occurs quite often in patients with insulin resistance, type 1 and even type 2 diabetes after insulin treatment and/or following intense exercise. Furthermore, hypoglycemia associated autonomic failure (HAAF) in diabetic patients is a serious condition in which antecedent hypoglycemia results in impaired autonomic counterregulatory responses (CRR). Therefore, identification of confounding factors that affect organization of a normal CRR is important.

Here, selective activation of arcuate orexigenic neurons and rapid increase of *Npy/Agrp* gene expression have been revealed in hypoglycemic mice. This effect was found to be specific for orexigenic neurons, since *Pomc* expressing medial basal hypothalamic (MBH) neurons do not express c-FOS in response to decrease in plasma glucose. These findings are consistent with those reported by Muroya *et al*.^11^ and Fioramonti *et al*.^12^. Arcuate NPY neurons play an essential role in driving counterregulatory responses to hypoglycemia because: (*1*) subset these cells are glucosensitive/glucose inhibited^12^; (*2*) *Npy* expression is rapidly upregulated in response to hypoglycemia^13^; (*3*) NPY fibers originating in the arcuate nucleus densely innervate preautonomic neurons in the hypothalamic paraventricular nucleus and intermediolateral cell column of the spinal cord, which provide sympathetic output to the liver, pancreas and adrenal medulla^14,15^ and (*4*) NPY mediates fasting-induced sympatho-adrenal synaptic plasticity and adrenaline release, which is important to maintain euglycemia^16^.

In addition to activation of hypothalamic NPY/AGRP neurons, microglia in the arcuate nucleus become activated in hypoglycemic animals as revealed by set of morphometrical measures in IBA1 stained material. First, activation of microglial cells was revealed by the increase of IBA1 stained area within the arcuate nucleus. Detailed spatial and morphological analysis showed that hypoglycemia elicited an increase of soma size and a decrease of the area occupied by microglia processes (solidity), corresponding to the accepted hallmarks of microglial responses to diverse activating stimuli^17^. Furthermore, an increase of the number of IBA1-ir profiles in hypoglycemic animals also implicates changes in microglial activity in response to physiological threat.

Activation of microglia following exposure emotional stressors is widely observed^18–20^, however, much less is known about the effects of physiological stressors on microglia^21^. Here, hypoglycemia-induced microglia activation was confined to the medial basal hypothalamus/arcuate nucleus/median eminence region and not seen in any other extrahypothalamic or limbic structures. By contrast, a study in rats revealed significant increase of CD11b positive microglia in the hippocampus following sustained and severe hypoglycemia (up to isoEEG), which was rather due to neuronal death following glucose reperfusion, but not to directly to the decrease of blood glucose^22^.

The critical involvement of microglia activation in hypoglycemia was assessed by minocycline, a second generation tetracycline, which inhibits microglia activation and has an anti-inflammatory action^19^. Based on morphological measures, minocycline administration attenuated insulin-induced microglia activation in the arcuate nucleus, which was accompanied with improved glycemic control, elevated plasma catecholamine levels and less severe hypoglycemia. This finding is consistent with reports of protective effects of minocycline on hypoglycemia-induced neuronal death and cognitive impairment^23^.

It is not likely that microglial cells are directly responsive to glucose deprivation, however, fasting and hypoglycemia results in acidic extracellular microenvironment, which might favor microglia activation through acid sensing receptors (ASICs and TDAG8)^21,24^. Furthermore, alarmins such as secreted extracellular heat shock protein (eHSP72)^25^, mtDNA fragments^26^ and HMGB1^27,28^ or ATP^9^ might be among the factors driving microglial sensitization/activation/sterile inflammation^29^ during hypoglycemia.

Confocal microscopic analysis of hypothalamic sections revealed that IBA1 positive microglia are recruited in close apposition to hypoglycemia-activated c-FOS positive, NPY neurons. This observation is comparable to that reported by Sugama *et al*. where microglial activation occurred adjacent to stress-responsive neurons in the periaqueductal gray (PAG) of restrained rats^30^. Such microglial arrangement suggests the involvement of signals originating in activated (c-FOS positive) neurons. Among neuronal signals which control microglia function, the role of fractalkine is emerging. Fractalkine is a CX_3_C chemokine expressed by neurons or activated astrocytes, which controls microglia executive functions upon binding to its unique receptor (CX_3_CR1) on microglia^10^. Indeed, CX_3_CR1^−/−^ mice with impaired neuron-to-microglia communication did not display overt microglia activation and did not develop hypoglycemia in response to insulin challenge. This is very likely due to their improved counterregulatory responses such as increased hypothalamic NPY/AGRP expression and sympathomedullary and adrenomedullary hormone secretion. Nonetheless, the role of additional neuronal factors regulating microglia activity, such as the complement factor C3 or CD200 might also play some role^31,32^.

In addition to hypothalamus, cells responding specifically to hypoglycemia have been found in the dorsal vagal complex, including the nucleus of the solitary tract, dorsal motor nucleus of the vagus and related circumventricular organ, area postrema^5,33^ as well as in catecholaminergic neurons of the ventrolateral medulla^34,35^. Because these areas also display c-FOS induction in hypoglycemic animals, activation of hindbrain microglia and their recruitment in glycemic control remains to be elucidated.

Among the factors released by activated microglia, interleukin IL-1 is major cytokine secreted upon physiological and/or stress challenges^8,21^. IL-1b causes hypoglycemia^36,37^ acting centrally to reset glucose homeostasis^38^. To support the involvement of IL-1 in modulation of hypoglycemia-induced counterregulatory responses, we showed that mice lacking both IL-1a and b (IL-1a/b KO) displayed less severe insulin-induced hypoglycemia than wild type controls.

It should be noted, however that microglia might not be the only central source of IL-1 in hypoglycemic stressed animals. There are reports showing reactive astrocytes and even activated neurons to be able to synthesize IL-1^39^. We showed an increase of GFAP+ profiles in ARC of insulin injected wild-type and fraktalkine receptor deficient mice (Supplementary Fig. 8). Furthermore, additional cytokines, such as TNFa and IL-6 might also be involved in hypoglycemia^37^. By contrast, we did not find significant increases of these cytokines at 1 hour time point at the mRNA level.

It should  also be noted that some of these effects might be due to parallel changes occurring at the periphery. For instance, hypoglycemia increases leukocyte counts and circulating levels of pro-inflammatory cytokines^40^, which act directly in the liver inhibiting gluconeogenesis^37^ or in the pancreas increasing endogenous insulin secretion. IL-1 increases glucose uptake and consumption at the periphery. Because both knockouts (CX_3_CR1^−/−^ and IL-1a/b KO) are not cell type specific, the reported microglia associated glucoregulatory changes are supplemented with actions mediated through peripheral monocytes and macrophages. Recently, fractalkine-CX_3_CR1 system has been implicated in the direct regulation of pancreatic islet function. Long acting fractalkine analogue reduced glucagon secretion and enhanced hepatic insulin sensitivity^41^. There is a possibility that differences in blood glucose levels seen in CX_3_CR1^−/−^ and IL-1a/b KO mice compared to WT following insulin administration are rather due to insulin resistance than impaired counterregulatory responses. To rule out this possibility, we performed insulin tolerance test in both genotypes and did not find overt differences in insulin sensitivity. We also used 2-DG, a non-metabolizing glucose analogue, which results in neuroglucopenia and provoke physiological counterregulatory responses. Importantly, both CX_3_CR1^−/−^ and IL-1a/b KO mice displayed improved glucoregulatory response to 2-DG as well, suggesting unrestrained CRR, not insulin insensitivity.

Collectively, our data show, for the first time, that acute hypoglycemia selectively activates hypothalamic microglia. These microglial cells are found in close apposition with orexigenic NPY/AGRP neurons in the arcuate nucleus. Attenuation of microglia activation results in improved counterregulatory responses to glucopenia and less severe insulin-induced hypoglycemia.

## Methods

### Experimental animals

All animal procedures were conducted in accordance with the European Communities Council Directive (86/609 EEC) and the Hungarian Act of Animal Care and Experimentation (1998; XXVIII, Sect. 243/1998), approved by the Animal Care and Use Committee of the Institute of Experimental Medicine, Hungarian Academy of Sciences (permit number: PEI/001/29-4/2013).

Experiments were performed on adult male C57BL/6 J, CX_3_CR1^−/−^ ^10^ CX3CR1^+/−^, IL-1 a/b KO^42^ and BAC-NPY/Cre//Gt (ROSA)26Sor_CAG/LSL_ ZsGreen1 mice (on C57BL/6 background).

Mice were group housed and maintained under controlled conditions (temperature: 22 ± 2 °C; humidity: 65%; lighting schedule: 07:00-19:00) with food and water available *ad libitum* unless otherwise stated.

### Insulin-induced hypoglycemia

Mice starved overnight (18 h) and injected intraperitoneally (i.p.) with 1IU/ml/kg bw insulin (Actrapid, Novo Nordisk). Blood glucose levels were measured by DCont Personal Blood Glucose Meter (77 Elektronika Kft. Hungary) from samples of trunk blood or tail snip. Control fed and fasted mice were injected i.p. with sterile saline.

### Intracerebroventricular (i.c.v.) injections

Mice were anesthetized with a cocktail of 100 mg/kg ketamine and 10 mg/kg xylazine (1 ml/100 g bw, i.p.) and the right lateral cerebral ventricle was approached by a stainless steel cannula at the following coordinates: A-P: −0,05 mm; L: −0,11 mm; D-V-0,2 mm. Mice were injected i.c.v. with 20 µg minocycline hydrochloride (M-9511, Sigma) in 2 µl saline. After injection, mice were fasted overnight and injected intraperitoneally (i.p.) with minocycline (50 mg/kg, 10 ml/kg) and with 0.8 IU/kg insulin 1 h before sacrifice.

### Glucoprivation

Male C57BL/6 J controls CX_3_CR1^−/−^, and IL-1 a/b KO mice were injected i.p. with 2-deoxyglucose (Sigma D8375; dose: 200 mg/kg bw) and were decapitated 1 h after injection for blood glucose analysis.

### Plasma hormone levels

Plasma corticosterone was measured by direct RIA as described^43^.

Plasma catecholamines (2-CAT, Labor Diagnostika Nord, Germany) and glucagon (Crystalchem, USA) were measured by ELISA according to the manufacturer’s instructions. For adrenaline and noradrenaline measurement extraction steps were performed before ELISA as instructed.

### Perfusion, tissue processing and immunohistochemistry

For histology, mice were anesthetized and perfused with ice cold fixative (4% paraformaldehyde in 0.1 M phosphate buffer pH 7.2). Four complete series of coronal sections (20 µm) were cut on freezing microtome and stored in cryoprotectant at −20 °C. Single-, double- and triple immunohistochemistry was done as described in^43^. Detailed protocols and analysis are found in the Supplemental Material.

*In situ hybridization histochemistry* was performed according to the protocols described in^44^ and modified by^45^ (for details see Supplementary Material).

### Imaging and c-FOS quantification

One complete series of sections (4 × 25 µm = 100 µm apart) were stained for each antigens. Digital images of hypothalamic paraventricular nucleus (PVN) (between bregma −0.58 and −0.94), and arcuate nucleus (ARC) as^46^ (between bregma −1.22 and −1.94) in C57BL/6 and CX_3_CR1^−/−^ mice were captured 20x magnification by Spot RT color digital camera (Diagnostic Instruments Inc., IL, USA) on Nikon Eclipse 6000 microscope. Images were then re-opened in Image J 1.48 software and set at a common threshold to subtract the background optical density. The software automatically counted the number of immunoreactive cell nuclei (c-FOS) within the region of interest (ROI). 3–4 sections per animal containing the ROI were used in the analysis (n = 3–5 mice).

### Analysis of microglia

To determine the area, the number, the distribution and morphological parameters of Iba1 + microglia, we used Microglia Analyzer, a MATLAB based, custom-designed software (K. Gulyás^43^). Quantification of Iba1+ cells were performed in the ROI using unit area square selection in the ARC or PVN area. The images were converted into a binary black-and-white format. An automated count of white pixels (representing all IBA1+ signals) was applied and the IBA1 staining was reported as percentage of unit area.

The convex area (CA) is the area of the smallest convex polygon drawn around the microglial cell by connecting the furthest points of its processes. The solidity index is calculated by dividing the area of the microglia (IBA1+) cell by its convex area (CA). The more ramified, thinner microglia, the bigger convex area is, thus yielding a smaller solidity index. Higher solidity values are characteristic of activated microglia, having thickened and shortened processes and smaller convex area (Supplementary Fig. 1).

Distribution of microglia in the ARC was characterized by the nearest neighbor distance (NND)^43^. For this calculation, the X-Y coordinates for each microglia were recorded at the region of interest. The Euclidean distance between each microglia and its nearest neighbor (NND) was calculated in Microglia Analyzer software. This value was then averaged for all images to define the value per animal.

### Quantitative real-time PCR

Total RNA was isolated from arcuate nucleus samples with QIAGEN RNeasyMiniKit (Qiagen, Valencia, CA, USA) according the manufacturer’s instruction. To eliminate genomic DNA contamination, DNase I (Fermentas) treatment was used. Sample quality control and the quantitative analysis were carried out by NanoDrop (Thermo Scientific). Amplification was not detected in the RT-minus controls. cDNA synthesis was performed with High Capacity cDNA Reverse Transcription Kit (Applied Biosystems, Foster City, CA, USA). Designed primers (Invitrogen) were used in real-time PCR reaction with Power SYBR Green PCR master mix (Applied Biosystems, Foster City, CA, USA) on ABI StepOnePlus instrument. The amplicon was tested by Melt Curve Analysis and mRNA levels were calculated by ABI StepOne 2.3 program using ΔΔC_T_ method. Experiments were normalized to GAPDH expression. Primer sequences are listed in Supplemental Material.

### Western blot

After decapitation, whole hypothalamic samples were dissected and immediately frozen at −70 °C. Total protein from tissue homogenates was isolated with Geneaid PrestoTM DNA/RNA/Protein Extraction Kit (Geneaid) according to the manufacturer’s instruction. Protein content was measured by PierceTM BCA Protein Assay Kit (ThermoFisher Scientific). After denaturation, samples were separated on 12% sodium dodecyl sulphate - polyacrylamide gel electrophoresis (SDS-PAGE). Immunoblotting was performed to polyvinylidene difluoride (PVDF) membranes (Millipore) using semi-dry transfer (Trans Blot SD Cell, Biorad). Non-specific binding was blocked in 5% BSA (Sigma) for 1 h. After incubation, PVDF membrane was cut between 42kDA and 17 kDa marks according to the protein ladder (Benchmark pre-stained protein ladder, Invitrogene). Membranes were incubated in anti-b-ACTIN (1:5000, Sigma) or rabbit anti-NPY (1:1000, courtesy Dr. R. Corder, Geneva Switzerland) primary antibodies, respectively at 4 °C overnight. This was followed by incubation in respective biotinylated secondary antibodies (1:1000, Vector Labs) and then in avidin-biotin-HRP complex (1:250, Vector Labs) for 1–1 h. Membranes were developed by immunoperoxidase reaction and analyzed by UVITec Q9 Alliance© software.

### Statistical analysis

Data are presented as mean ± standard deviation (SD) and were analyzed by one-way ANOVA (in case of three different groups) or two-way ANOVA with Bonferroni post hoc test using GraphPad Prism software (ver. 6.01; San Diego, CA, USA). On the figures, * marks genotype difference, # marks treatment effects. To analyze differences between two group means, unpaired or paired two sided t-test was performed. In all cases, p-value < 0.05 was considered statistically significant.

## Supplementary information

Supplementary material

## Data Availability

The datasets generated during and/or analyzed during the current study are available from the corresponding author on reasonable request.
