# Supplementary material for: Hypoglycemia-activated Hypothalamic Microglia Impairs Glucose Counterregulatory Responses
Source: Sci Rep. 2019 Apr 17;9:6224. doi: 10.1038/s41598-019-42728-3 (PMC6470310; doi:10.1038/s41598-019-42728-3)

Hypoglycemia-activated Hypothalamic Microglia Impairs Glucose Counterregulatory Responses

Zsuzsanna Winkler[1,2], Dániel Kuti[1,2], Ágnes Polyák[1], Balázs Juhász[1,2], Krisztina Gulyás[1], Nikolett Lénárt[1,3], Ádám Dénes[1,3], Szilamér Ferenczi[1] and Krisztina J. Kovács[1]

1. Laboratory of Molecular Neuroendocrinology, Institute of Experimental Medicine, Budapest, Hungary,

2. János Szentágothai Doctoral School of Neurosciences, Semmelweis University Budapest, Hungary,

3. Laboratory of Neuroimmunology, Institute of Experimental Medicine, Budapest, Hungary.

Corresponding author:

Krisztina J. Kovács, PhD

Laboratory of Molecular Neuroendocrinology

Institute of Experimental Medicine,

Budapest, Hungary.

phone: +36-1-210-9952

Email: kovacs@koki.hu

Supplementary Information

Insulin tolerance test

Following overnight fast, insulin (1.0 IU/ml/kg body weight, Actrapid, Novo Nordisk) was injected intraperitoneally to adult, male C57BL/6J (wild-type) and $CX_3CR1^{-/-}$ mice. Immediately before and then at different time points (15, 30, 60, 240 min) after insulin administration, mice were decapitated to collect trunk blood samples and to measuring blood glucose levels with D-Cont Personal Blood Glucose Meter (77 Elektronika Kft. Hungary).

Glucose tolerance test

Adult, male C57BL/6J (wild-type) and $CX_3CR1^{-/-}$ mice were fasted overnight (15 h) and then injected intraperitoneally with 2 mg/g of body weight D-glucose. Blood glucose was measured from tail vein by D-Cont Personal Blood Glucose Meter at 0 min (just before glucose injection) and at 15-, 30-, 60-, and 120-min intervals after glucose load.

Single-, double- and triple-label immunohistochemistry

Immunohistochemical detection of c-FOS (rabbit polyclonal 1:10000, sc-52, Santa Cruz Biotechnology) or ionized calcium-binding adaptor molecule 1, IBA-1 (rabbit polyclonal 1:500, 019-19741, WAKO) was performed according to the standard avidin-biotin-HRP protocol using Ni-ammonium sulphate-intensified diaminobenzidine (DAB) chromogen.

Double label immunohistochemistry: To identify c-Fos positive neurons and Iba-1 positive microglia on the same sections, c-Fos immunostaining has been performed first with DAB-Ni intensification, followed by Iba-1 staining with DAB chromogen only.

Immunofluorescence:

NPY-ir profiles were detected in the medial basal hypothalamus by rabbit anti-NPY antibody (1:1000, courtesy R. Corder, Geneva, Switzerland) using donkey anti rabbit Alexa594 (1:500, A21207 Life Technologies).

To characterize hypoglycemia-activated neurons in the lateral hypothalamus, c-FOS staining was combined with Orexin (goat anti-orexin A, 1:2000 sc-8070, Santa Cruz) or Melanin-concentrating hormone, MCH (goat anti-MCH, 1:1000, sc-14509 Santa Cruz) immunofluorescence using donkey anti rabbit Alexa594 (1:500, A21207 Life Technologies), and donkey anti goat Alexa488 (1:500, A11055 Life Technologies).

Sections from BAC-NPY/Cre//Gt (ROSA)26Sor_CAG/LSL_ ZsGreen1 mice were stained with c-FOS (rabbit polyclonal 1:10000, sc-52, Santa Cruz Biotechnology) or ionized calcium-binding adaptor molecule 1, Iba-1 (rabbit polyclonal 1:500, 019-19741, WAKO) and donkey anti rabbit Alexa594 (1:500, A21207 Life Technologies) to reveal activation of NPY neurons in response to hypoglycemia and apposition between NPY neurons and microglia, respectively.

In hypothalamus, GFAP immunoreactivity was visualized by mouse anti-GFAP monoclonal antibody (1:2000, G3893 Sigma Aldrich) using donkey anti mouse Alexa594 (1:500, A21203 Life Technologies) secondary antibody.

Immunofluorescent triple labeling was performed on tissue samples obtained from $CX_3CR1^{+/gfp}$ microglia reporter mice using c-Fos and non-phosphorylated neurofilament H (NF-H) (mouse 1:500, BioLegend) antibodies, to visualize microglia (GFP), activated neuronal cell nuclei (c-FOS) and neurons (NF-H), respectively. The antigens were then visualized by biotinylated horse anti-mouse IgG (Vector Labs, 1:500) for 1 hour followed by streptavidin Alexa 405 (Molecular Probes, 1:500) and donkey anti-rabbit IgG conjugated with Alexa Fluor 594 (Invitrogen, 1:500) for 3 hours. After washing, sections were mounted on slides and covered with Fluoromount-GTM Solution (Southern Biotechnology Associates).

Fluorescent images were taken by confocal laser scanning using Nikon C2+ microscope. Appositions of GFP positive microglial processes and c-FOS positive neurons were evaluated by using Z-stack imaging. Three-dimensional image analysis was carried out by using NIS-Elements Viewer 4.2 software.

In situ hybridization histochemistry

^{35}S-UTP (NEG039H Perkin Elmer) -labeled antisense cRNA probes were generated from cDNA fragments corresponding to proopiomelanocortin, POMC (0.3 kb 1-337, courtesy Dr. J. Eberwine) and proNPY (0.3kb, exon2 fragment, courtesy Dr. D. Larhammar) following linearization of pBluescript plasmids and using T3 (POMC) and T7 (NPY) RNA polymerases.

When c-FOS immunostaining was combined with in situ hybridization histochemical detection of NPY or POMC mRNA, immunostaining was performed first, according to a modified protocol described by Chan et al. [13] and the reaction product was developed without nickel enhancement. Then, sections were mounted on Superfrost Ultra+ (Menzel Gläser) slides and processed for in situ hybridization as described [12, 13].

Densitometric analysis of integrated densities of the *in situ* hybridization histochemistry was performed using the ImageJ software on scanned images obtained by the BASReader program (Raytest Isotopenmessgerate GmbH, Straubenhardt, Germany). Anatomical regions were identified on the captured images using the mouse brain atlas [14]. The density measurement was corrected by subtraction of a background value obtained from a neighboring non-hybridized tissue area from the same section. Measurements over the arcuate region were taken bilaterally from each slice.

Quantitative real-time PCR

Primer sequences for the following genes are:

gene	forward primer	reverse primer
Gapdh	TGACGTGCCGCCTGGAGAAA	AGTGTAGCCCAAGATGCCCTTCAG
c-fos	GACAGCCTTTCCTACTACCATTCC	GGACAGATCTGCGCAAAAGTC
Npy	CAGATACTACTCCGCTCTGCGACACTACAT	TTCCTTCATTAAGAGGTCTGAAATCAGTGTCT
Agrp	AGGACTCGTGCAGCCTTACAC	AGCTTTGGCGGCGGTGCTA
Pomc	CGAGATTCTGCTACAGTCGCTCAGG	GCCAGGAAACACGGGCGTCT
Iba-1	AGCTGCCTGTCTTAACCTGCATC	TTCTGGGACCGTTCTCACACTTC
Il-1a	CCATAACCCATGATCTGGAAGAG	GCTTCAGTTTGTATCTCAAATCAC
Il-1b	CTCGTGGTGTCGGACCCATATGA	TGAGGCCCAAGGCCACAGGT
Il-6	TCCGGAGAGGAGACTTCACA	TGCAAGTGCATCATCGTTGT
Tnf-a	CAGACCCTCACACTCAGATCA	GGCTACAGGCTTGTCACTCG
Ccl2	CCAGCACCAGCACCAGCCAA	TGGATGCTCCAGCCGGCAAC
Ccl5	CAGCAGCAAGTGCTCCAATCTT	TTCTTGAACCCACTTCTTCTCTGG
Ikb	ACTTAAGTCCCGACCCTCCA	AAGCACAAAGCAAAGCACCC
Il-1ra	CTTTACCTTCATCCGCTCTGAGA	TCTAGTGTTGTGCAGAGGAACCA

Supplementary Figure 1. **Image analysis of Iba-1 positive microglial cells.**

A: Selection of the region of interest (ROI) in the hypothalamic arcuate nucleus using the rectangle selection tool. 3V is the third ventricle. *B*: The number of Iba-1 positive microglia, automatically quantified by Microglia Analyzer, a MATLAB-based software in the selected area (the numbering of identified glia cells are shown in yellow). *C*: Result of segmentation with Microglia Analyzer software. *D*: Convex area (CA, yellow) of one microglial cell. CA defines the area of the convex polygon formed by connecting the furthest point of microglial processes. Scale bars represent 20 μm.

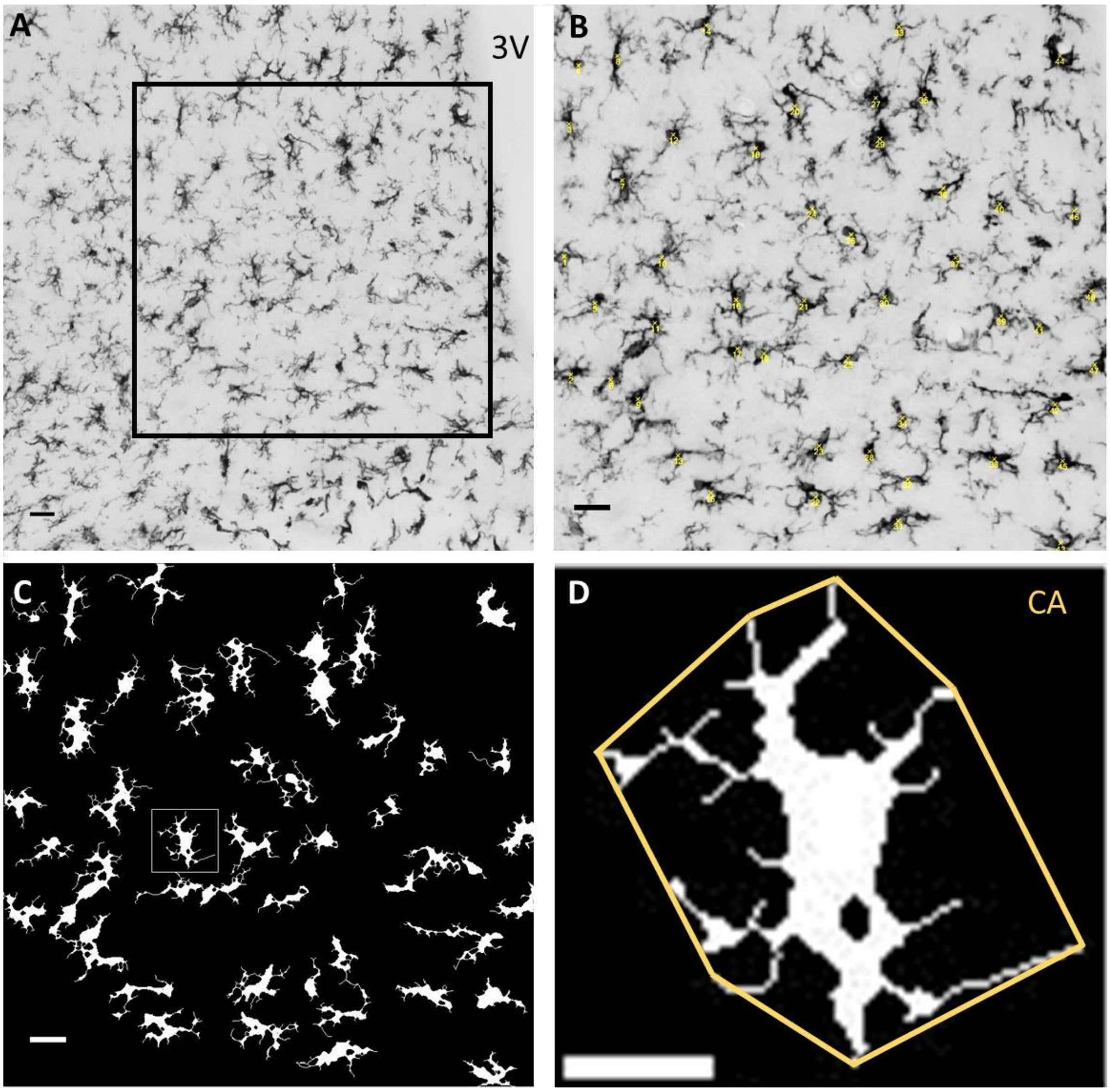

Supplementary Figure 2. **Representative images of NPY immunoreactivity in the arcuate nucleus**

ROI are shown on images. Fed: fed, saline-injected group; Fasted: O/N fasted, saline-injected group; Fasted+insulin: O/N fasted, insulin-injected group. Scale bars, 20 μm; 3V: third ventricle, ME: median eminence

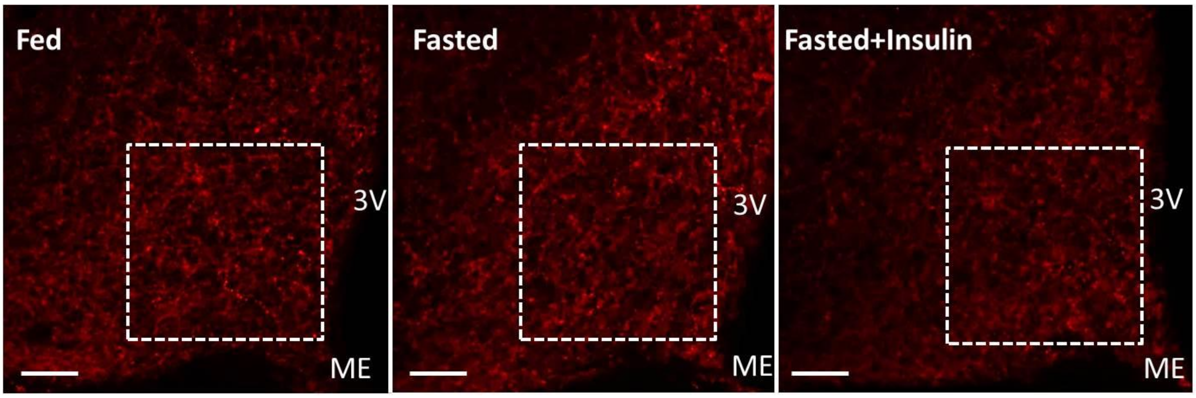

Supplementary Figure 3. **Representative image of full-length NPY and ß-ACTIN Western blot from hypothalamus of Fed, Fasted, Fasted+insulin treated C57Bl/6 (wild-type) mice**

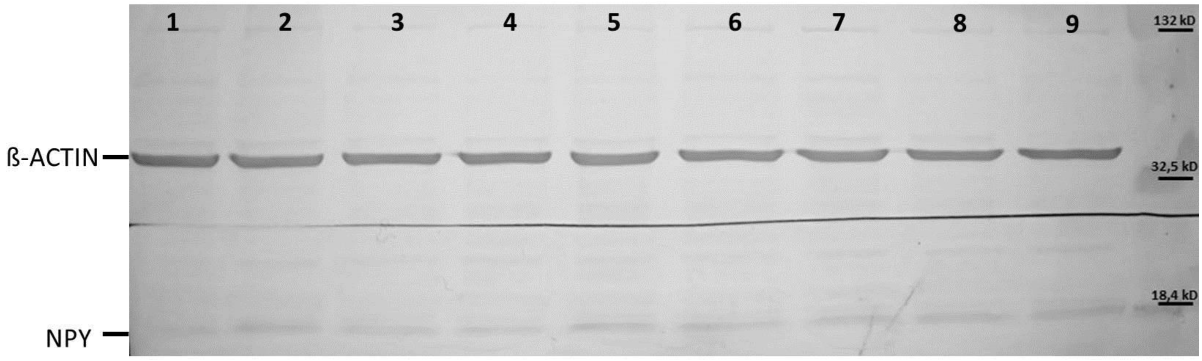

Lanes: Fed: 1,2, 3; Fasted: 5,7,9; Fasted+Insulin: 4,6,8

Supplementary Figure 4. O**rexin-A and MCH in the lateral hypothalamus (LH)**

A-*B*: Low- (scale bars, 100 µm) and high magnification (scale bars, 25 µm) images of c-FOS (red) and OREXIN-A (green) (*A*) or MCH peptide (green) (*B*) colocalization in LH of C57BL/6 mice following insulin-induced hypoglycemia. 3V: third ventricle, f: fornix. *C*: Number of OREXIN-A positive cells in the lateral hypothalamus (n=3 per groups). *D*: Counts of MCH positive cells (mean±SEM) in the lateral hypothalamus (n=3 per groups). Fed: fed, saline-injected group; Fasted: O/N fasted, saline-injected group; Fasted+insulin: O/N fasted, insulin-injected group.

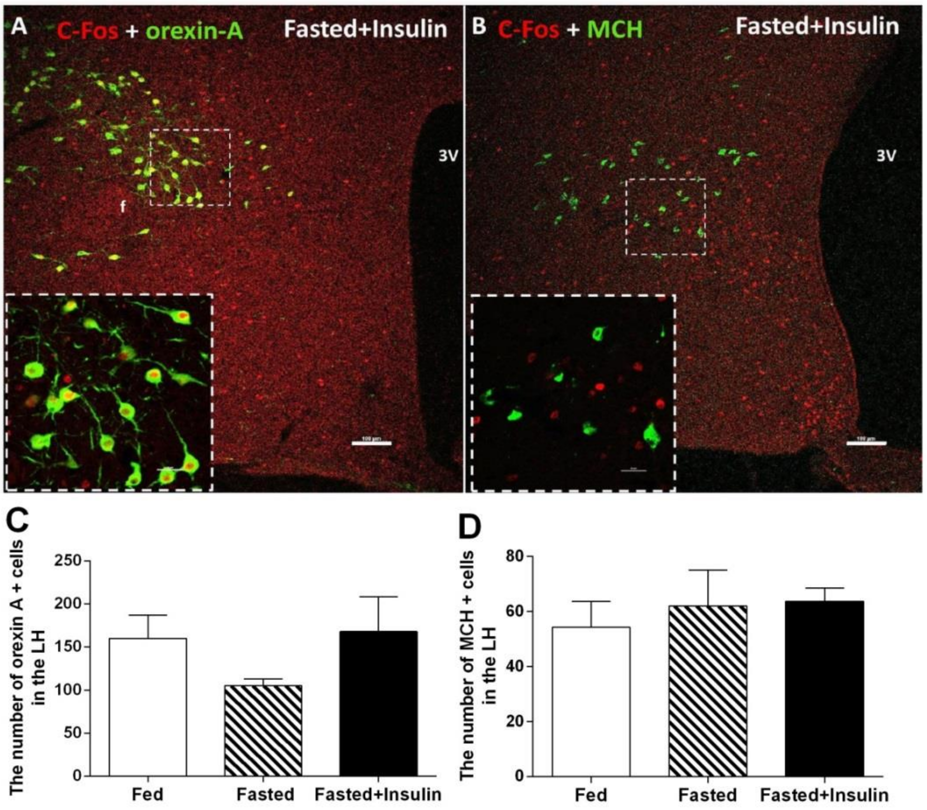

Supplementary Figure 5. **Qualitative and quantitative analysis of ionized calcium-binding adaptor molecule 1 (IBA1) immunostained microglia in the paraventricular nucleus of the hypothalamus (PVN).**

A: Photomicrographs, showing IBA1 immunoreactive microglia in the PVN of fed, vehicle injected and hypoglycemic C57BL/6 mice (fasted+insulin). Scale bars, 20 μm. *B*: Mean±SEM percentage of the area covered by IBA1-immunoreactivity per unit area in the PVN (n= 3 per group).

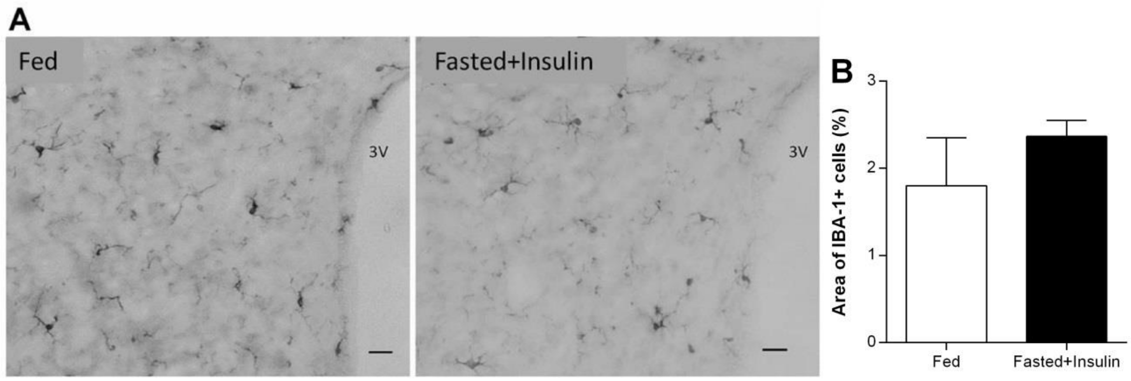

Supplementary Figure 6. **Microglia in the cortex and hippocampus/dentate gyrus (DG) of fasted+insulin injected mice (pre)treated with minocycline or vehicle (icv).**

A,*D*: IBA1 immunoreactive microglia in the cortex (*A*) and hippocampus, dentate gyrus (*D*)., Mean±SEM values of the areas covered by IBA1 immunoreactive profiles (*B* and *E*) and the number of IBA1 positive microglial cells (*C*, *F*) (n=4 per groups).

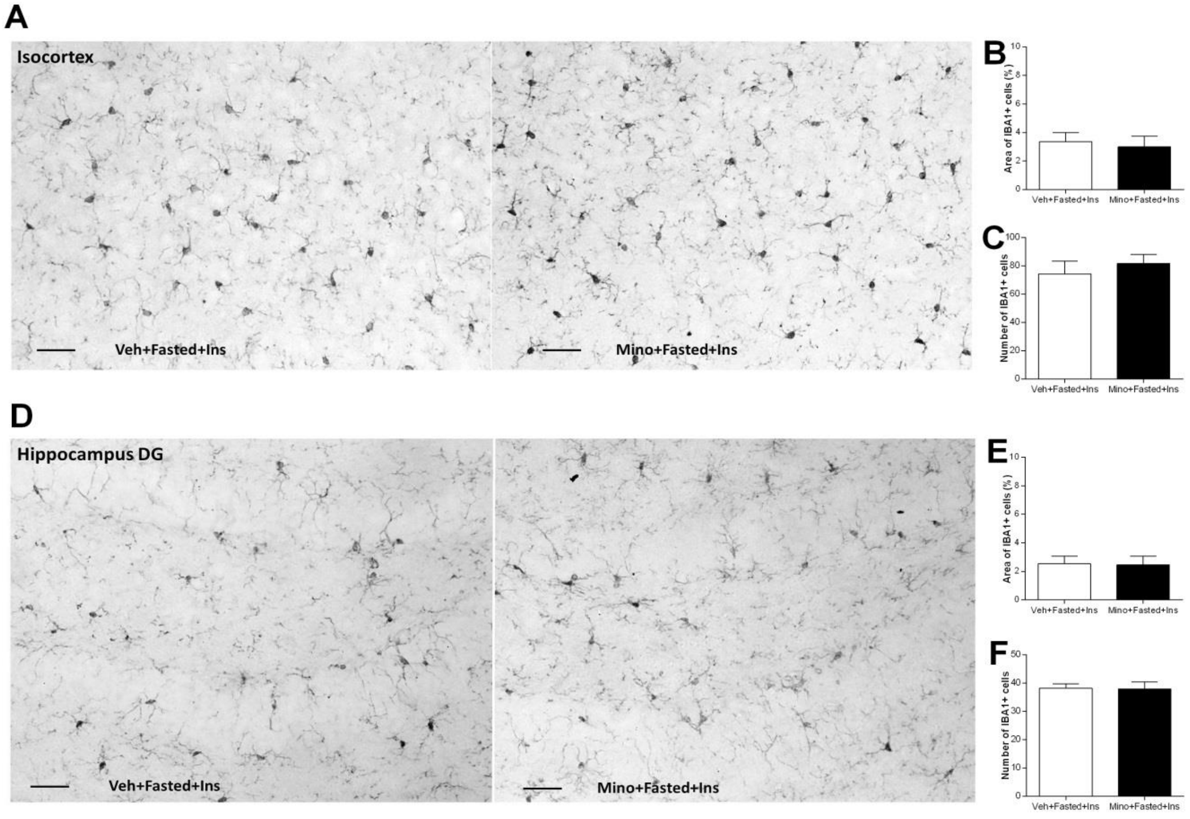

Supplementary Figure 7. **Glucose tolerance test in fractalkine receptor deficient mice (CX3CR1 -/-) and wild-type mice.**

Blood glucose levels were measured after O/N fasting at time point 0 and 15-, 30-, 60-, and 120-min following i.p. injection of 2 mg/g bw glucose.. Data expressed as mean±SEM (n=5 per genotype).

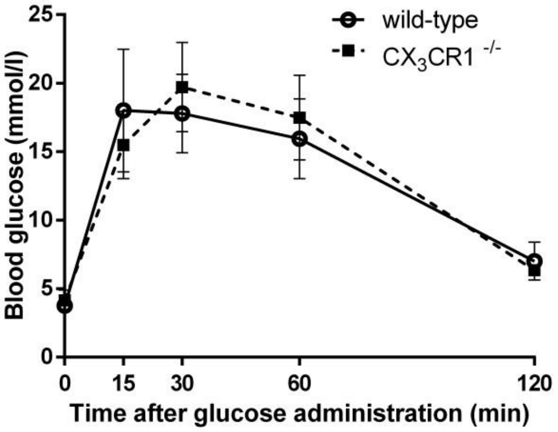

Supplementary Figure 8. **Astrocytes in arcuate nucleus after hypoglycemia**

A: Representative images of astrocyte marker, GFAP, (white) in the medial basal hypothalamus of fed, vehicle injected and hypoglycemic mice. ROI are shown on images. Scale bars, 20 μm; 3V: third ventricle, ME: median eminence. *B*: Pixel density of GFAP-immunreactivity in ARC, in wild-type and fractalkine receptor deficient mice (CX3CR1 -/-) (n= 3 per groups). Fed: fed, saline-injected group; Fasted+insulin: O/N fasted, insulin-injected group. Data expressed as mean±SEM. Two-way ANOVA revealed significant treatment effect indicated by ##p<0.01.

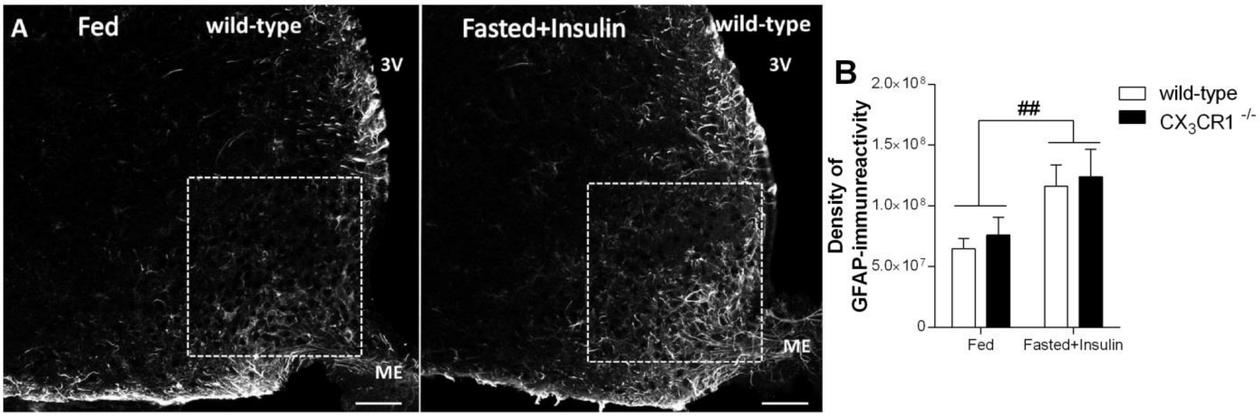

Supplement: Supplementary file 1 — Supplementary material [file 41598_2019_42728_MOESM1_ESM.pdf]
